# Supplementary material for: The cardiac microenvironment uses non‐canonical WNT signaling to activate monocytes after myocardial infarction
Source: EMBO Mol Med. 2017 Aug 3;9(9):1279–93. doi: 10.15252/emmm.201707565 (PMC5582413; doi:10.15252/emmm.201707565)
Supplement: Supplementary file 1 — Appendix [file EMMM-9-1279-s001.pdf]

Appendix

TABLE OF CONTENTS

Legends to Appendix Figures 2
Appendix Supplementary Methods 3
Appendix Figures 5
Appendix Tables 8

Legends to Appendix Figures

Appendix Figure S1) Confirmation of major RNAseq hits in monocytes isolated from the BM compared to monocytes isolated from the heart by qPCR. Major RNAseq hits of the canonical WNT pathway (Lrp1, Dab2) and the non-canonical WNT pathway (Fos, Jun) could be confirmed by qPCR with a similar X-fold increase.

Appendix Figure S2) Flow cytometric analysis of inflammatory monocytes demonstrate an increased expression of ROR2 in the heart compared to bone marrow monocytes.

Appendix Figure S3) Relative luciferase intensity in raw macrophages stimulated with supernatant from normoxic or hypoxic HL-1 cardiomyocytes.

Appendix Figure S4) WIF1 expression in human tissue sections of the left ventricle from individual patients two to four days after acute myocardial infarction (MI, top panel) and from patients with endstage heart failure (HF, bottom panel). Blue: DAPI, Red: WIF1. White arrows indicate prominent WIF1 expression.

Appendix Figure S5) Baseline cardiac function of adult WIF1 KO animals compared to control mice.

Appendix Figure S6) Mortality rate of WT and WIF1KO animals after induction of MI. A total of 106 animals mice were included (52 WIF1KO and 54 WT animals).

Appendix Figure S7) Expression of non-canonical WNT components in monocytes isolated from the heart of WT and WIF1KO animals three days after MI.

Appendix Figure S8) Inflammatory monocytes (left) and macrophages (right) 4 days after induction of myocardial infarction in bone marrow transplanted animals.

Appendix Figure S9) Validation of AAV- and adenovirus-mediated WIF1 overexpression *in vivo* (top) and *in vitro* (bottom).

Appendix Figure S10) Baseline cardiac function of WIF1 overexpressing mice compared to control mice.

Appendix Figure S11) Purity control for sorted inflammatory monocytes.

Appendix Figure S12) Gating strategy for cell sorting of inflammatory monocytes.

Appendix Supplementary Methods

Bone marrow transplantation

Mice were irradiated with 9Gy in one dose. Irradiated mice received 1x106 bone marrow cells from WT or WIF1KO animals via i.v. injection. Donor and recipient mice were sex and age matched. Bone marrow was allowed to be reconstituted for 4 weeks.

Histological sampling and analysis

Hearts were extracted on the indicated time points. A slice of the left ventricle from the point of the LAD-ligation and half the distance to the apex was dissected and prepared for paraffin embedding. Apex was kept for biochemical and molecular biological analysis.

Sections of approx. 50µm beneath the LAD-Ligation were discarded. Consecutive sections were mounted on Superfrost Plus microscope slides (Thermo Scientific, Braunschweig, Germany).

At least 3 sections per animal were histologically analyzed in a double blinded manner.

Monocyte sorting

Heart and bone marrow of WIF1 and WT animals at the age of 14 weeks were extracted 3 days after induction of MI. Bone marrow cells and minced hearts were digested as described in the method section. Single cell suspensions of digested heart and bone marrow cells were incubated with conjugated PE-antibodies directed against lineage markers (see method sections). Cells were washed and incubated with anti-PE-magnetic particles (BD Biosciences). Negatively selected cells were further incubated with anti-CD11b magnetic particles (BD Biosciences). RNA of positively selected cells were isolated using TRIZOL.

ATF2 reporter assay

RAW 264.7 were transfected with 50 ng ATF2-firefly-luciferase (affymetrix) using Lipofectamine® LTX (Life technologies). RAW 264.7 cells were then stimulated for 4h with supernatant of HL-1 cells that were cultured under normoxic or hypoxic conditions for 24h.

Cells were lysed and Luciferase Assay Substrate (Promega) in Luciferase Assay Buffer (Promega) was injected. Bioluminescence was measured on FLUOstar Omega multi-mode microplate reader. Data were not normalized.

Appendix Figures

S1

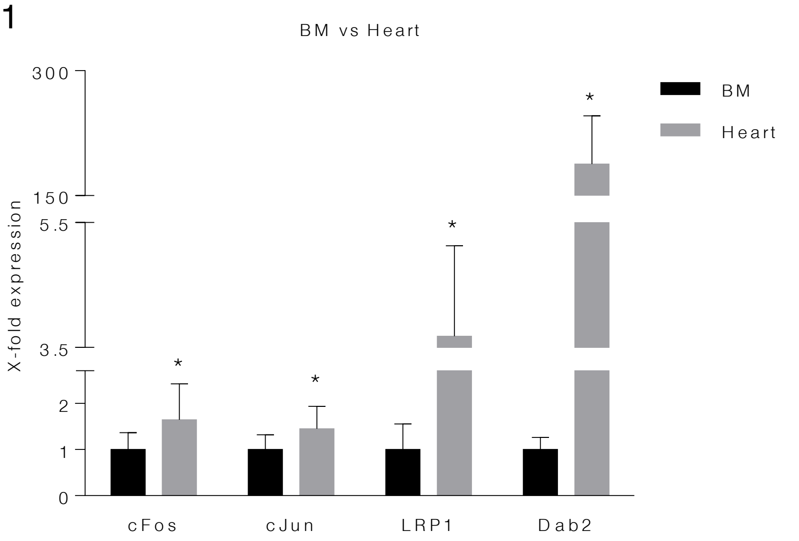

S3

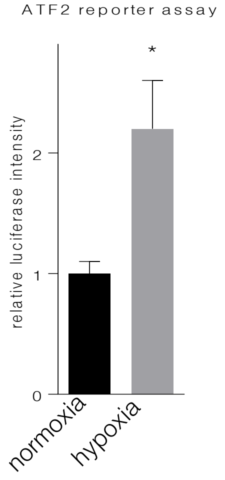

S2

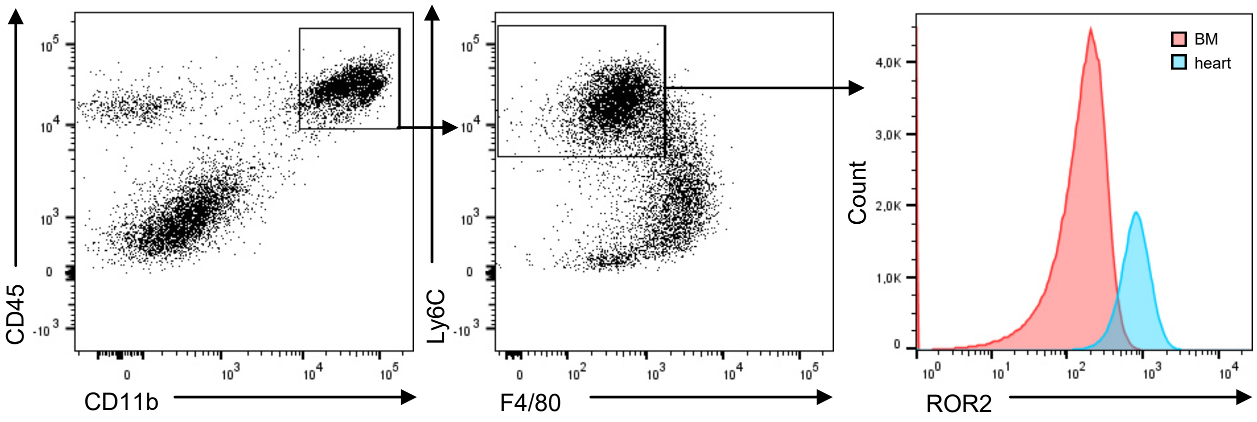

S4

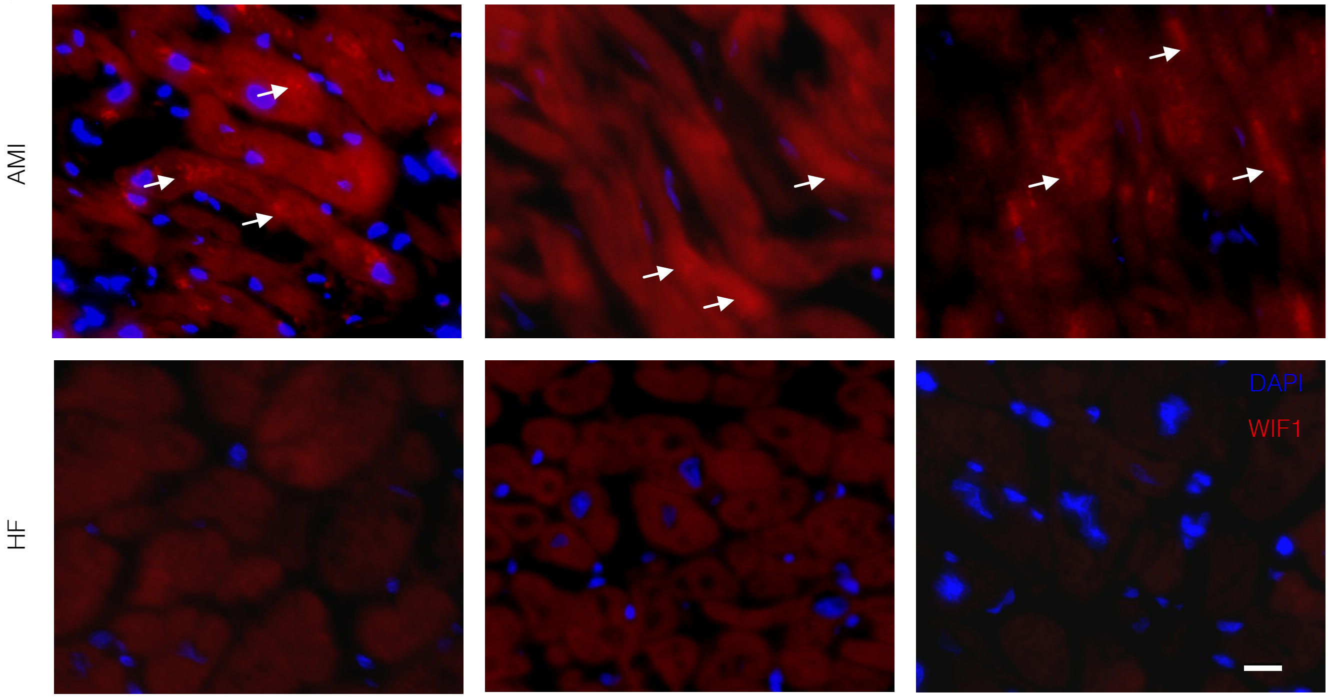

S5

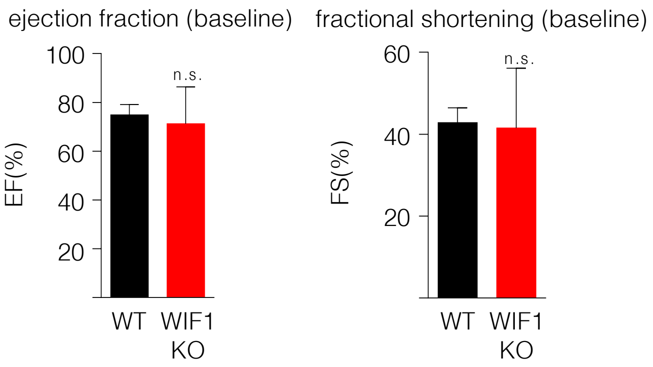

S6

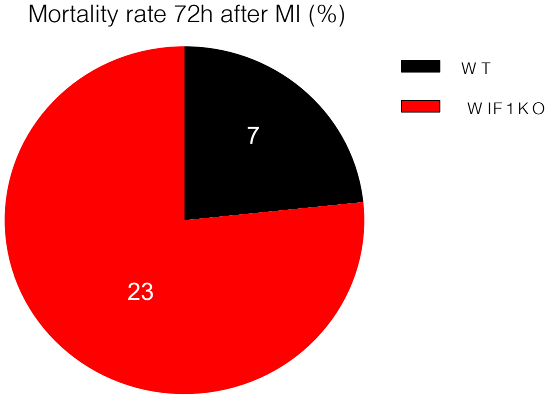

S7

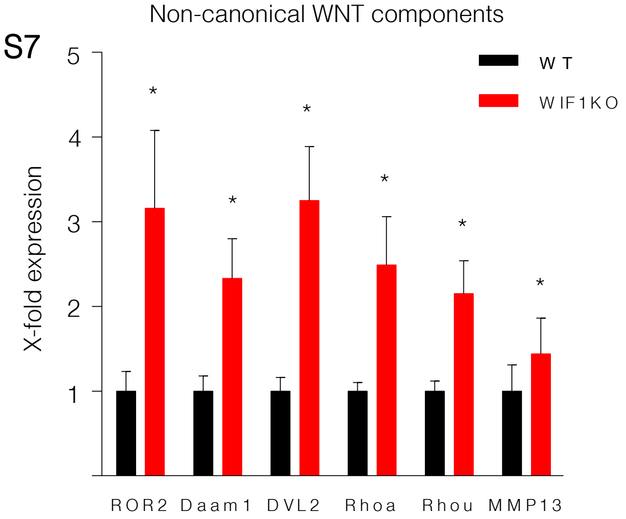

S8

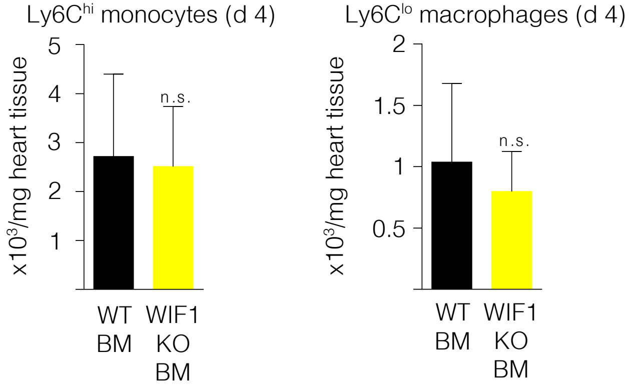

S9

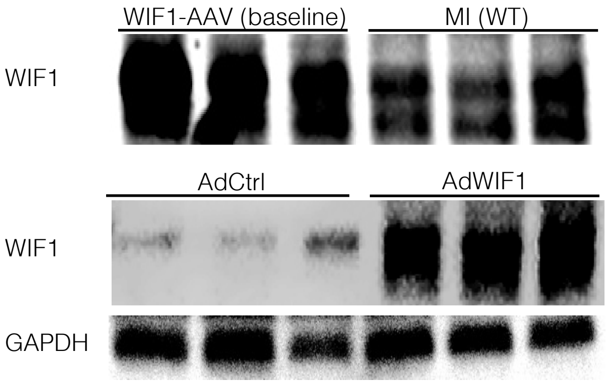

S10

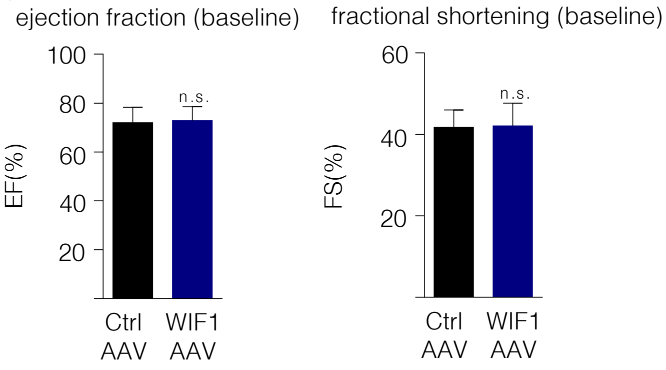

S11

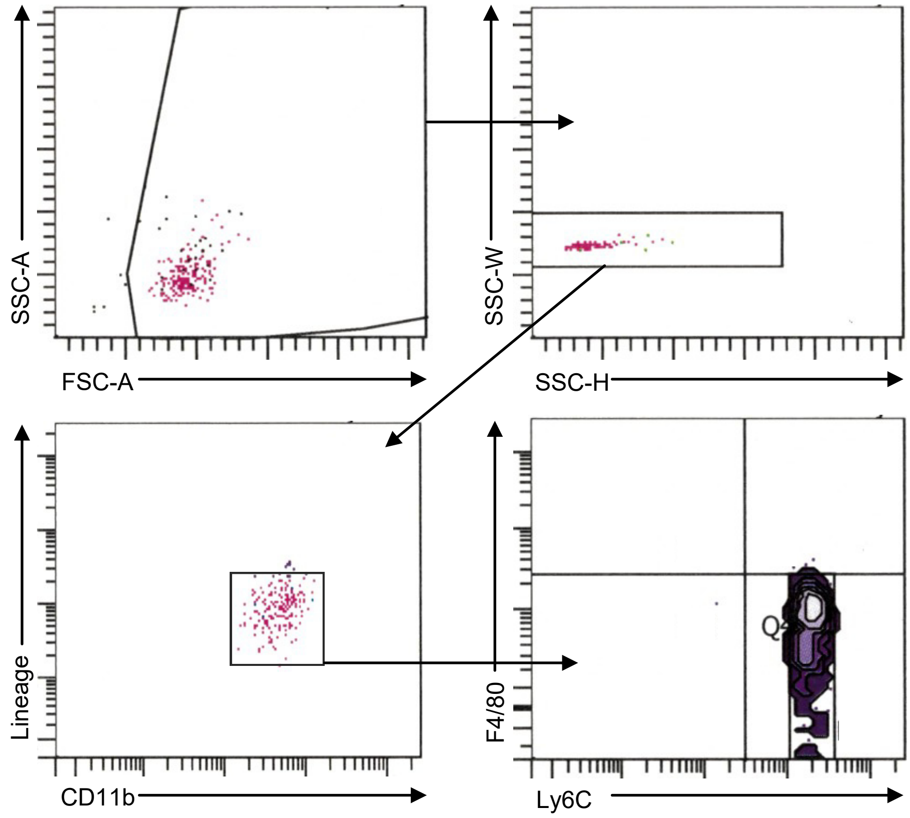

SSC-A
FSC-A
SSC-W
SSC-H
Lineage
CD11b
F4/80
Ly6C

S12

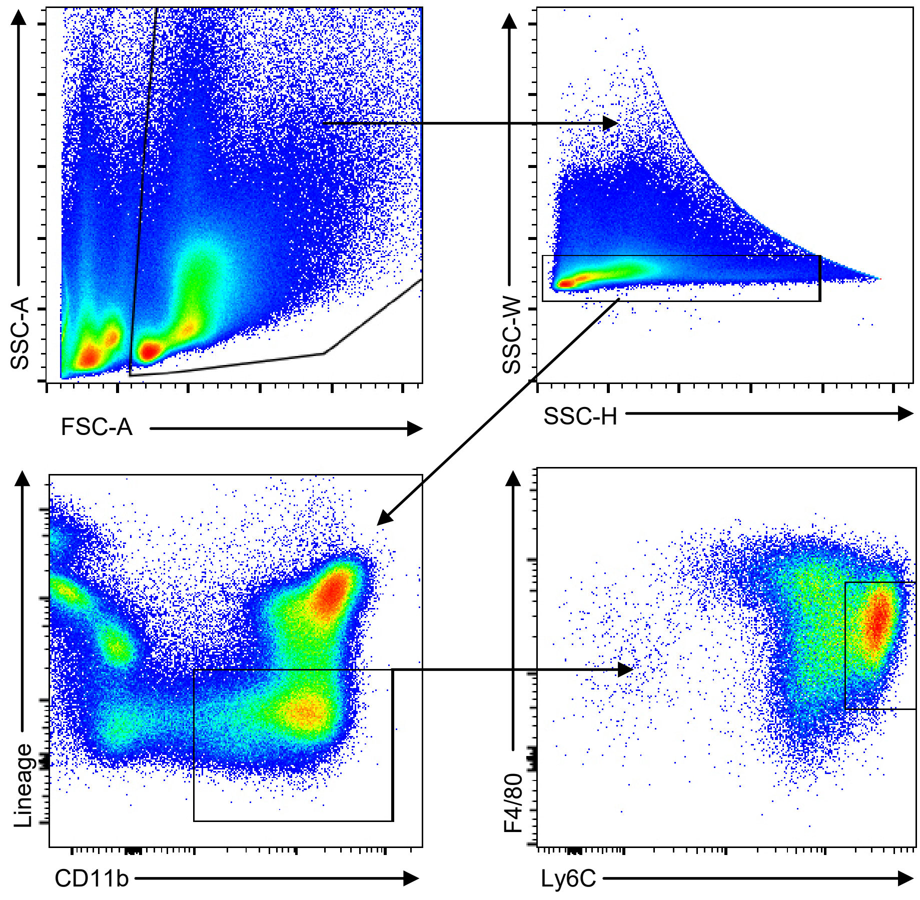

SSC-A
FSC-A
SSC-W
SSC-H
Lineage
CD11b
F4/80
Ly6C

Appendix Tables

ID (binding motif)	test.p.value	test.p.adj	
GGGAGGRR_V$MAZ_Q6	3,23842E-13	2,65227E-10	
AACTTT_UNKNOWN	3,80509E-12	1,55818E-09	
TGANTCA_V$AP1_C	5,15251E-11	1,40664E-08	non-canonical WNT transcription factor
CAGGTG_V$E12_Q6	4,71827E-10	7,33639E-08	
CTTTGT_V$LEF1_Q2	4,7625E-10	7,33639E-08	canonical WNT transcription factor
TGGAAA_V$NFAT_Q4_01	5,37464E-10	7,33639E-08	
V$OCT1_B	1,59414E-07	1,86514E-05	
GCANCTGNY_V$MYOD_Q6	1,83924E-07	1,88292E-05	
V$OCT1_01	3,57312E-07	3,25154E-05	
TTGTTT_V$FOXO4_01	6,22969E-07	4,63829E-05	
YATGNWAAT_V$OCT_C	5,74307E-07	4,63829E-05	
V$HMEF2_Q6	1,71304E-06	0,000100213	
GTGGGTGK_UNKNOWN	1,62506E-06	0,000100213	
CTAWWWATA_V$RSRFC4_Q2	1,58436E-06	0,000100213	
V$AP1_01	2,10749E-06	0,000109901	
AATGTGA,MIR-23A,MIR-23B	2,14703E-06	0,000109901	
V$OCT_Q6	2,73309E-06	0,000130752	
GATAAGR_V$GATA_C	2,87368E-06	0,000130752	
CTGCAGY_UNKNOWN	3,42851E-06	0,000147787	
V$SRF_Q6	3,90434E-06	0,000159883	
V$FOXD3_01	7,86933E-06	0,000292954	
TATAAA_V$TATA_01	7,75143E-06	0,000292954	
V$AREB6_03	9,20017E-06	0,000301398	
CTGAGCC,MIR-24	8,61914E-06	0,000301398	
TTANTCA_UNKNOWN	8,91163E-06	0,000301398	
V$TATA_01	1,01487E-05	0,000304405	
V$HFH4_01	1,01487E-05	0,000304405	
RTAAACA_V$FREAC2_01	1,0407E-05	0,000304405	
V$OCT1_03	1,30055E-05	0,000367293	
V$ATF3_Q6	1,44418E-05	0,000394262	
V$RSRFC4_01	1,73903E-05	0,000431595	
V$CEBP_Q2_01	1,6934E-05	0,000431595	
TGASTMAGC_V$NFE2_01	1,71811E-05	0,000431595	
V$COREBINDINGFACTOR_Q6	2,08567E-05	0,000502401	
V$AP1_Q6_01	2,5597E-05	0,00059897	
CAGCTG_V$AP4_Q5	2,78689E-05	0,000634018	
V$CHOP_01	3,81565E-05	0,000833673	
TGACCTY_V$ERR1_Q2	3,86808E-05	0,000833673	
V$NRF2_Q4	4,06705E-05	0,000854081	
V$OCT_C	4,53432E-05	0,000928403	
V$SOX9_B1	4,93437E-05	0,00098567	

Appendix Table S1. C3 motif gene sets significantly enriched (with $p < 0.001$) in inflammatory monocytes sorted from blood and heart.

	WT	WIF1KO	p
**cTNT**	5809±388	6084±450	0.65
**FS**	15.59±3.79	11.07±4.51	0.02
**EF**	32.39±6.84	23.54±9.02	0.02
**scar size**	39±5	61±11	0.01
**HW/BW**	6.52±0.73	7.95±1.50	0.02

Appendix Table S2. Summary of heart function parameters in WT and WIF1KO mice four weeks after MI.

	LUC-AAV	WIF1-AAV	p
**cTNT**	13487±781	12033±735	0.19
**FS**	9.94±1.95	23.42±3.58	0.01
**EF**	21.38±8.99	46.38±13.67	0.01
**scar size**	46±12	25±12	0.05
**HW/BW**	7.39±1.68	5.96±0.87	0.04

Appendix Table S3. Summary of heart function parameters in AAV-LUC and AAV-WIF1 injected mice four weeks after MI.
