# Supplementary figures and images for: The cardiac microenvironment uses non‐canonical WNT signaling to activate monocytes after myocardial infarction
Source: EMBO Mol Med. 2017 Aug 3;9(9):1279–93. doi: 10.15252/emmm.201707565 (PMC5582413; doi:10.15252/emmm.201707565)

# Figure 2A: Day 1

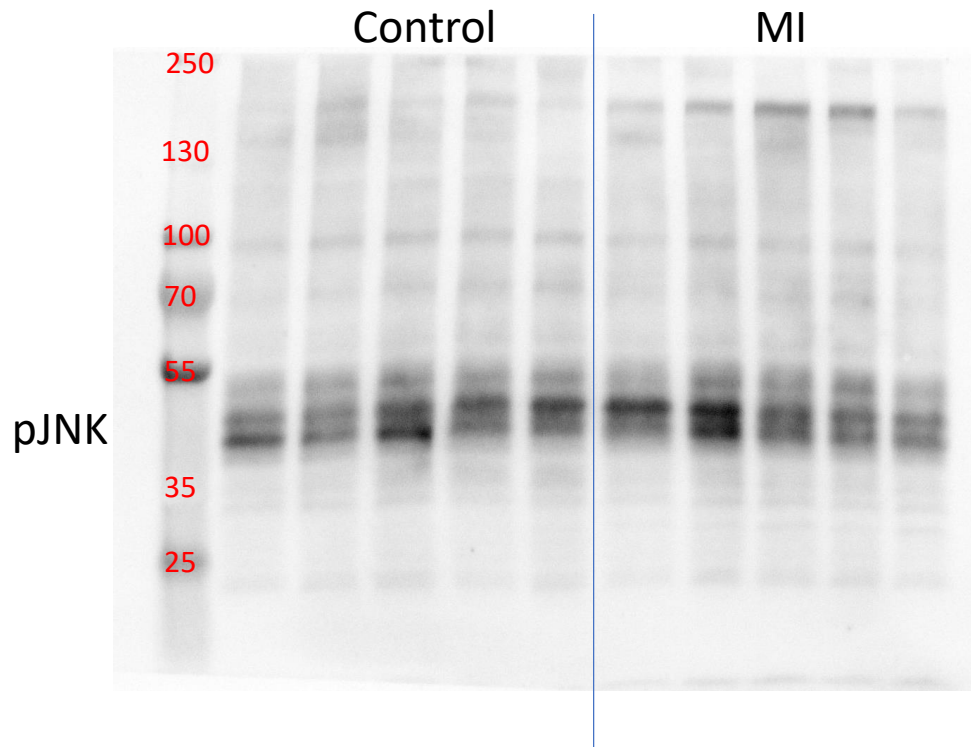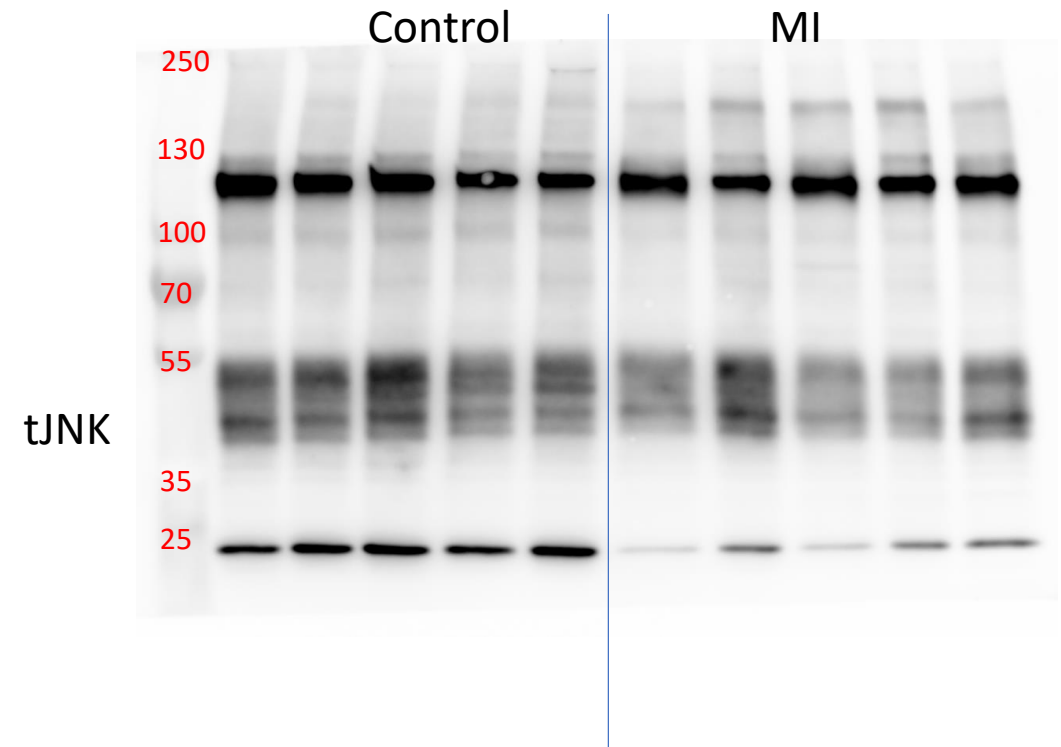

# Figure 2A: Day 2

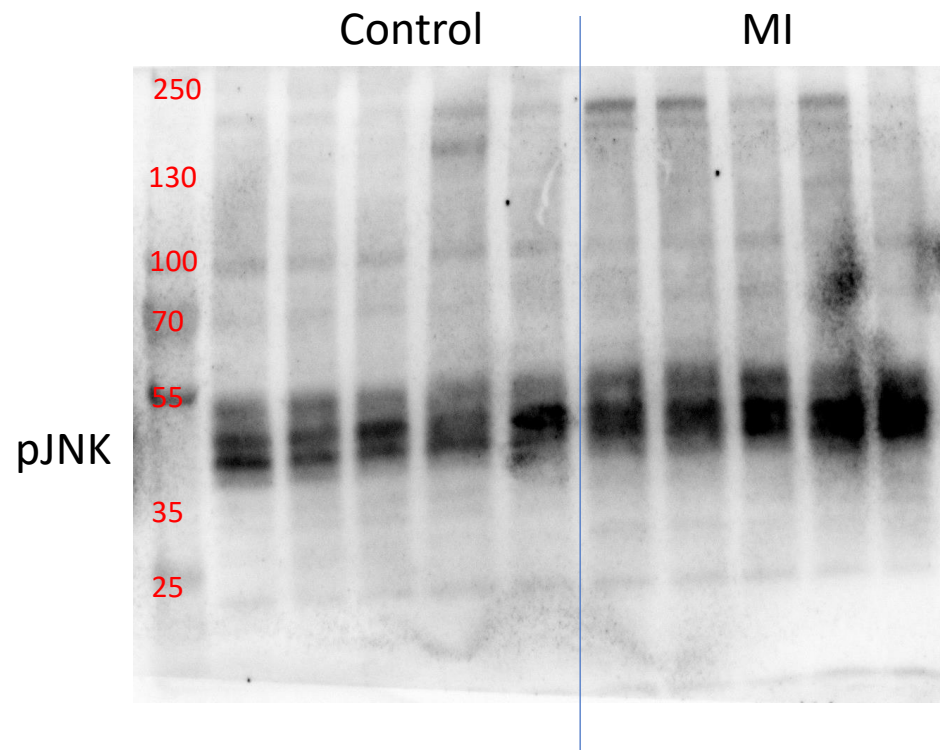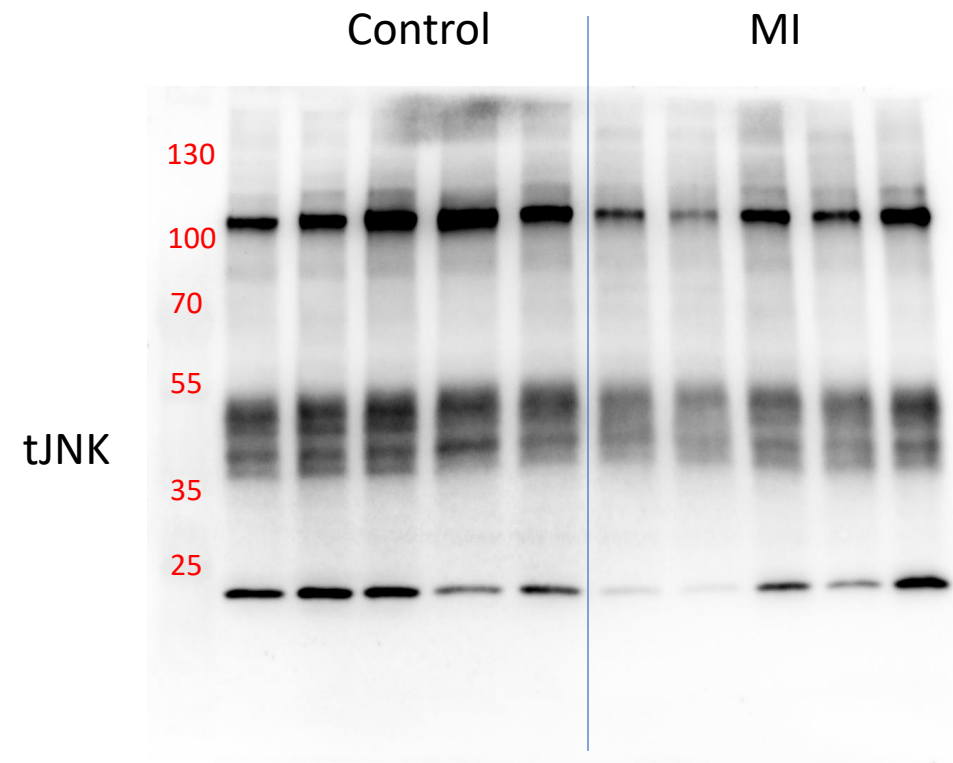

# Figure 2A: Day 2

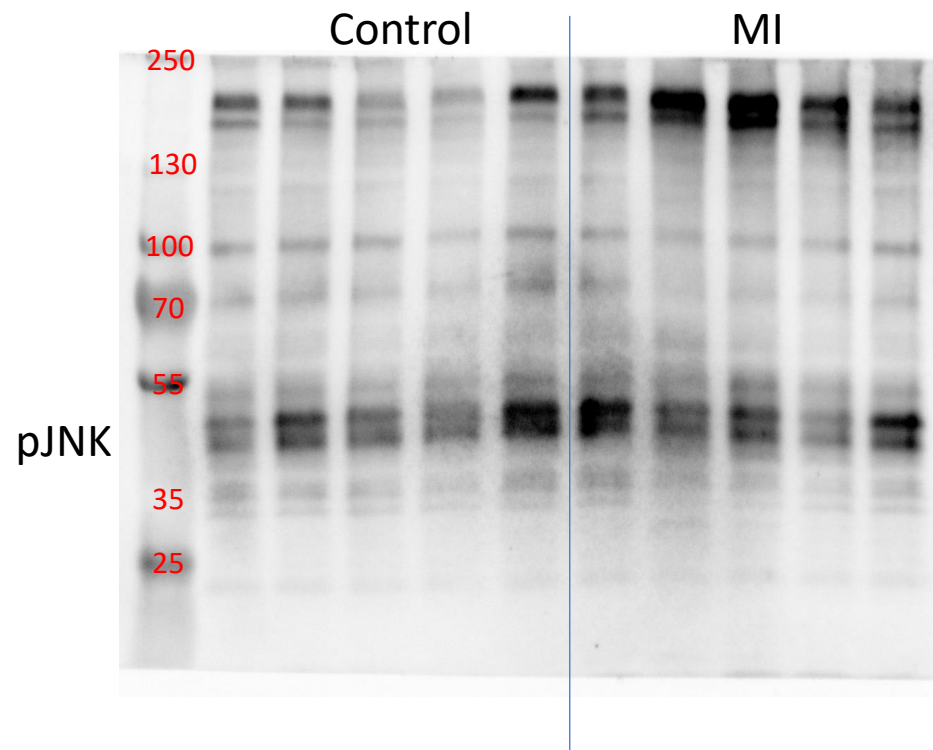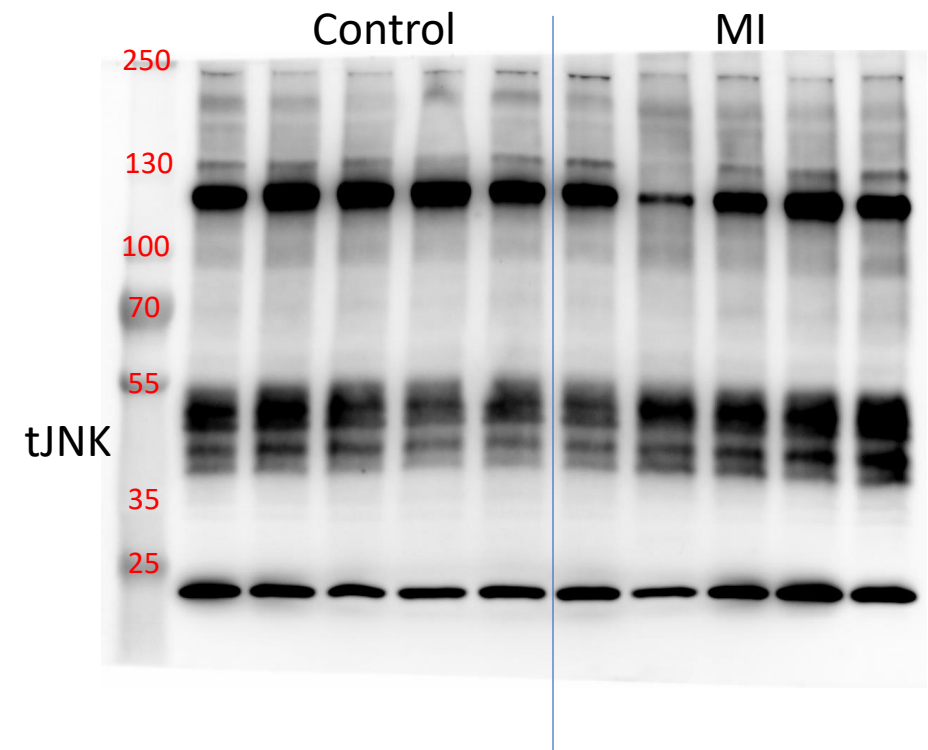

# Figure 2c

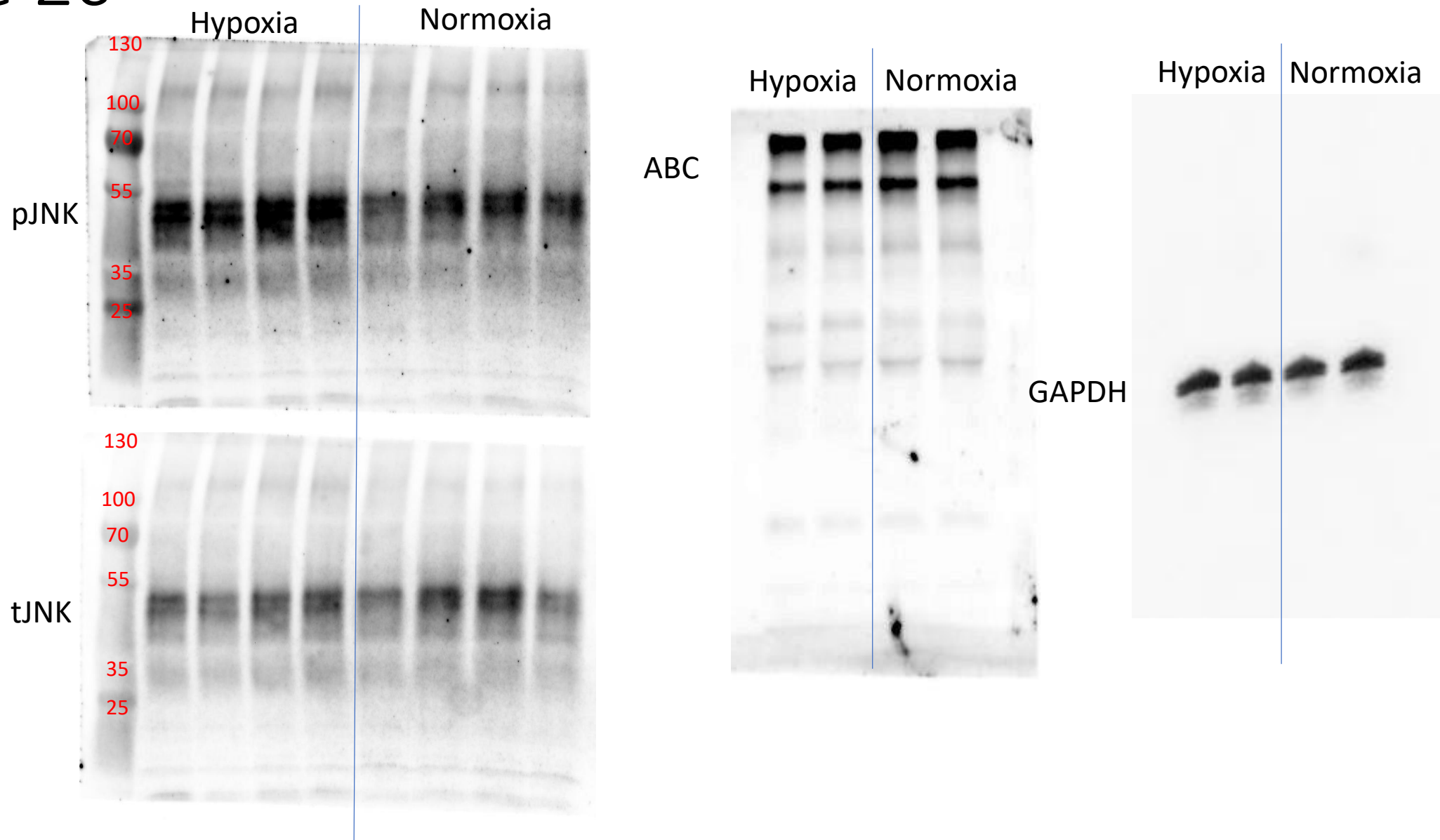

Supplement: Supplementary file 3 — Source Data for Figure 2 [file EMMM-9-1279-s002.pdf]

Figure 3

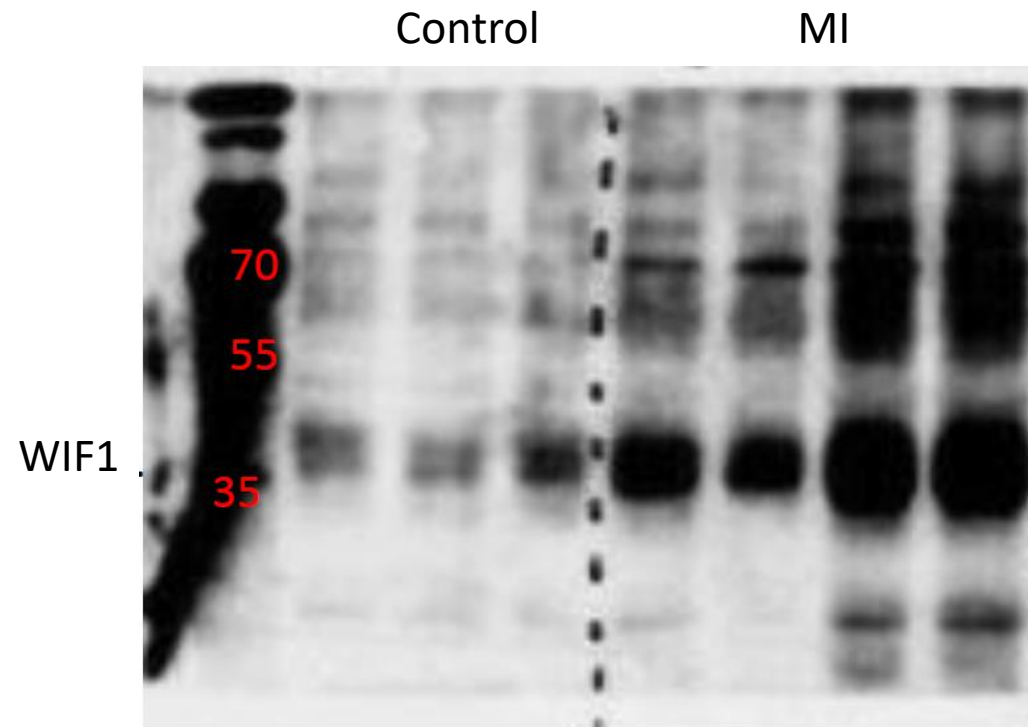

Supplement: Supplementary file 4 — Source Data for Figure 3 [file EMMM-9-1279-s003.pdf]

# Figure 6C

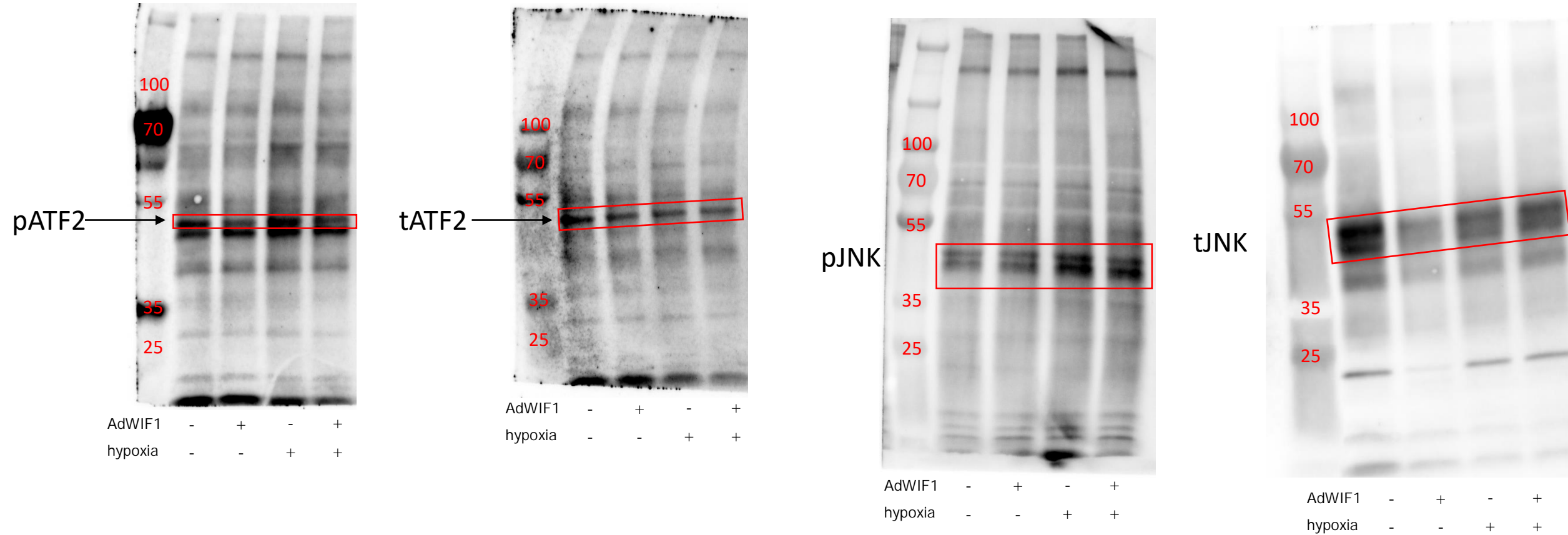

Supplement: Supplementary file 5 — Source Data for Figure 6 [file EMMM-9-1279-s004.pdf]
